# Localized vs. Systematic Neurodegeneration: A Paradigm Shift in Understanding Neurodegenerative Diseases

**DOI:** 10.3389/fnsys.2017.00062

**Published:** 2017-08-22

**Authors:** Armin Bayati, Taryn Berman

**Affiliations:** Department of Neuroscience, University of Victoria Victoria, BC, Canada

**Keywords:** localized neurodegeneration, neurodegenerative diseases, systematic neurodegeneration, Parkinson's disease (PD), Huntington's disease, schizophrenia, motor neuron disease, spinocerebellar degenerations

The categorization of neurodegenerative disorders have yet to be undertaken with regards to their mechanistic progression and their characteristic patterns of degeneration (Przedborski et al., 2003). Most of the categorization in this field is done with regards to patient's symptoms, their behavioral presentations, and deterioration (Reetz et al., 2010; Weintraub, 2011; Blesa et al., 2012; Rohrer et al., 2015; Eberhardt and Topka, 2016). Neurodegeneration and neurodegenerative diseases are one of the most researched and yet the most mysterious disorders in neuroscience. Their popularity is attributed to their ability to cause significant deterioration in behavior within short periods of time, ultimately impairing quality of life, and in some cases shortening life expectancy (Hardiman et al., 2016). Patients with neurodegenerative diseases are essentially given no hope, with no chances of healing, regression of symptoms, or regeneration of the atrophied brain areas conceivable (Wood-Kaczmar et al., 2006). Neurodegenerative diseases often have genetic causes, but repetitive traumatic brain injuries have also been associated with developing neurodegenerative diseases, as in the case of chronic traumatic encephalopathy (Stein et al., 2015). Medications have largely allowed the controlling of symptoms and an increase in the quality of life for inflicted patients, but no targeting of what causes such disorders have been attempted until very recently (Chaudhuri et al., 2006; Kirkeby et al., 2017). The purpose of this article is to suggest very basic, yet fundamental, ways in which neurodegenerative diseases can be categorized. This method of categorization can lead to a significant paradigm shift of neurodegenerative disorders by the public and scientific community. In short, it would allow for neuroscientists to set clear objectives as to what their goal should be in their prospective research areas when considering neurodegeneration.

The concept of localization of function within regions of the telencephalon and the diencephalon has been a major goal in the neuroscientist community (Stelzer et al., 2014). Localization of function allows for better treatments of neurological disorders and for more certainty to be attained regarding the nature and mechanism with which these disorders manifest (Lee et al., 2010; Seidel et al., 2010). Localized neurodegeneration is a label posed by this paper to characterize neurodegenerative disorders that cause dysfunction, atrophy and apoptosis to specific regions of cells in the brain. Localized neurodegeneration has yet to be used as a term to describe such disorders of the nervous system, and it is this lack of categorization that leads to the ambiguity of the nature of these neurological disorders. There are numerous diseases related to localized dysfunction of cells within the brain: Parkinson's Disease, Huntington's Chorea, frontotemporal lobar degeneration, spinocerebellar ataxia, spinomuscular ataxia, and motor neuron diseases (Burvill, 2009; Obeso et al., 2010; Finkbeiner, 2011; van Gaalen et al., 2011; Hamilton and Gillingwater, 2013; Res et al., 2015). Therefore, localized neurodegenerative diseases have a place of origin with causes being traced to a single localized event, and through regenerating the region of origin the afflicted individual can essentially be cured (Cheng et al., 1996; Elliott Donaghue et al., 2014; Kim et al., 2014; Siddiqui et al., 2015). For example, in Parkinson's Disease the replacement of dopaminergic cells in the substantia nigra would restore stimulation to the basal ganglia, theoretically resulting in a decline of the patient's dyskinesia and related movement abnormalities; however, this only holds true provided the integrity of the basal ganglia nuclei are intact (Zhang et al., 2014).

Opposing the category of localized neurodegenerative disorders, and their pathological mechanisms, are systematic neurodegenerative disorders. These are disorders that involve the interaction and breakdown of multiple systems. With these disorders the regeneration of any one region of the brain would not serve as a lasting treatment for the afflicted individuals (Hardy et al., 1992; Herminghaus et al., 2003; Hardy, 2006; Reif et al., 2006; Howes and Kapur, 2009). Systematic neurodegeneration presents a key unifying factor among all the affected cells, which allow for the possibility of these affected cells to be targeted. For instance, in schizophrenia various localized regions of the frontal and the prefrontal cortex are known to be affected and undergo degeneration (Gupta and Kulhara, 2010). Despite the known mechanism of the disease the cause of the disorder, while still under debate, is hypothesized to result from a systematic overactivation of dopamine receptors in the mesolimbic and mesocortical pathways (Howes and Kapur, 2009). This implies that the patient would not be treated via regeneration of the affected region since dopamine overstimulation would remain. Similarly, in Alzheimer's disease the regeneration of the atrophied brain areas would not serve as a viable form of treatment, since atrophy does not cause the disease, but results from it (Uchida, 2010). This indicates that Alzheimer's disease results from a systematic problem within the central nervous system and regeneration of cerebral tissue would not prevent amyloid plaques from forming (Hardy, 2006).

Being able to differentiate between these different types of neurodegenerative disorders allows for simple, explicit, objectives for medical and research professionals in developing treatments for these disorders. Such a paradigm shift is required for the right approach to be taken by medical researchers seeking to find more appropriate treatments for neurodegenerative diseases. In localized neurodegeneration, the regenerative approach can be taken by replacing the damaged region. In systematic degeneration the agent in which causes the degeneration, for instance overtransmission of specific neurotransmitters in the case of schizophrenia, should be addressed and its effects should either be deterred or counteracted using symptom targeted treatments.

## Author contributions

All authors listed, have made substantial, direct and intellectual contribution to the work, and approved it for publication.

### Conflict of interest statement

The authors declare that the research was conducted in the absence of any commercial or financial relationships that could be construed as a potential conflict of interest.
